# Association between Increased the De Ritis Quotient and Renal Azotaemia in Canine Babesiosis

**DOI:** 10.3390/ani12050626

**Published:** 2022-03-02

**Authors:** Olga Gójska-Zygner, Justyna Karabowicz, Justyna Bartosik, Wojciech Zygner

**Affiliations:** 1 Labros Veterinary Clinic, Św. Bonifacego 92, 02-940 Warsaw, Poland; olgazygner@yahoo.pl; 2 Division of Parasitology and Parasitic Diseases, Department of Preclinical Sciences, Institute of Veterinary Medicine, Warsaw University of Life Sciences—SGGW, Ciszewskiego 8, 02-786 Warsaw, Poland; justyna_karabowicz@sggw.edu.pl (J.K.); justyna_bartosik@sggw.edu.pl (J.B.)

**Keywords:** aspartate aminotransferase, azotaemia, *Babesia canis*, canine babesiosis, de Ritis quotient, renal index

## Abstract

**Simple Summary:**

This study investigated the association between increased serum aspartate aminotransferase (AST) activity and renal indices such as renal failure index, sodium fractional excretion, and urinary creatinine to serum creatinine ratio in dogs with renal azotaemia infected with *Babesia canis*. This research revealed that increased serum AST activity in azotaemic dogs may be of renal origin, and the de Ritis quotient may be a convenient and useful tool in the recognition of renal azotaemia in canine babesiosis.

**Abstract:**

Previous studies of azotaemia in canine babesiosis revealed pre-renal and renal azotaemia in infected dogs, and an association between an increased de Ritis quotient (aspartate aminotransferase to alanine aminotransferase activity; AST/ALT ratio) and azotaemia in affected animals. Serum activities of AST and ALT, and AST/ALT ratio were compared between azotaemic and non-azotaemic dogs infected with *Babesia canis*, and between affected dogs with pre-renal and renal azotaemia. Statistical analyses revealed higher AST activity and an increased AST/ALT ratio in azotaemic dogs, and an increase of these two parameters in infected dogs with renal azotaemia in comparison to dogs with pre-renal azotaemia. Moreover, AST activity and AST/ALT ratio were correlated with renal indices such as renal failure index, sodium fractional excretion, and urinary creatinine to serum creatinine ratio. The study also revealed a lack of correlation between AST and ALT activities in azotaemic dogs, although a correlation was observed when including all dogs in this study (azotaemic and non-azotaemic dogs treated as one group). The results of this study indicate that increased serum AST activity in azotaemic dogs infected with *B. canis* may have a renal origin, and the AST/ALT ratio could be considered as a simple and convenient renal index that is useful in the recognition of renal azotaemia in canine babesiosis.

## 1. Introduction

Canine babesiosis is a tick-borne protozoan disease caused by infection with parasites of the genus *Babesia*. There are at least six species of the parasite that infect dogs: *Babesia canis*, *B. rossi*, *B. vogeli*, *B. gibsoni*, *B. vulpes*, and *B. conradae* [1,2,3]. Infection causes mild to severe forms of the disease. It may lead to anemia and azotaemia, kidney, liver, and cardiac injury, various endocrine disorders, pancreatitis, septic shock, and even death [4,5,6,7,8,9,10].

Previous studies have revealed that azotaemia in canine babesiosis may be pre-renal or renal depending on the severity and duration of the disease [11,12]. It has also been shown that an increase in the de Ritis quotient (ratio of aspartate aminotransferase to alanine aminotransferase activity; AST/ALT ratio) in *B. canis*-infected dogs is associated with azotaemia, suggesting renal involvement in the elevation of serum AST activity and the de Ritis quotient in affected dogs [13]. The purpose of this preliminary study was to test the hypothesis that an increased de Ritis quotient in canine babesiosis results from kidney injury.

## 2. Materials and Methods

### 2.1. Study Sample

Diagnosis of babesiosis was made in 39 dogs of various breeds and age during a one-year period. There were 21 male and 18 female dogs. Preliminarily diagnosis of infection was based on microscopic blood smear examination. In dogs included in this research, the infection was confirmed by a PCR method described in detail in a previous study [14]. Moreover, absence of infection by *Anaplasma phagocytophilum* (the cause of the second most prevalent tick-borne infection in dogs and various other mammals in Poland) was confirmed using a previously described PCR method [15,16,17]. Exclusion criteria were as follows: other known disease before infection or diagnosed co-infection with *A. phagocytophilum*. Five dogs (four females and one male) were excluded from the study. Two dogs had other chronic diseases prior to *Babesia* infection (diabetes mellitus and hypothyroidism), and two dogs were infected with *A. phagocytophilum* and *B. canis* concurrently. One dog that did not survive babesiosis was not included in the study owing to anuria, which made the collection of urine samples impossible. Thirty-four dogs infected with *B. canis* were included in this research. Blood and urine samples were collected from dogs during the initial visit to the clinic, prior to any treatment.

### 2.2. Renal Indices

Serum activities of AST and ALT, and concentrations of serum urea, and serum and urine creatinine were determined using a clinical chemistry analyzer (Erba XL 640, Erba Diagnostics, Mannheim, Germany). Serum and urine sodium concentrations were determined using another clinical chemistry analyzer (EasyElectrolytes, Medica, Bedford, MA, USA). The obtained results were used to calculate the de Ritis quotient and indices of renal damage, such as the renal failure index (RFI), sodium fractional excretion (FE(Na^+^)), the serum urea to creatinine ratio (SU/SCr), and the urinary creatinine to serum creatinine ratio (UCr/SCr) [18,19].

### 2.3. Groups and Subgroups of Dogs

The obtained results allowed for separation of the dogs into two groups: A (azotaemic dogs) and B (non-azotaemic dogs). Dogs included in group A had concentrations of serum urea or creatinine, or both, at levels above reference intervals. Dogs included in group B had concentrations of both these parameters either within or below reference intervals (i.e., reference interval for serum urea concentration: 20–45 mg/dL; reference interval for serum creatinine concentration: 1.0–1.7 mg/dL). Dogs from group A were subdivided into two subgroups: A1 (dogs with pre-renal azotaemia) and A2 (dogs with renal azotaemia). The type of azotaemia was recognized using previously mentioned indices of renal damage: RFI, FE(Na^+^), SU/SCr, and UCr/SCr. If at least three of these four parameters indicated pre-renal azotaemia, the dogs were included into subgroup A1. If at least three indices of renal damage indicated renal azotaemia, the dogs were included into subgroup A2. RFI < 1, FE(Na^+^) < 1%, SU/SCr ≥ 20 (using urea and creatinine mg/dL), and UCr/SCr > 40 indicated pre-renal azotaemia. RFI > 2, FE(Na^+^) > 2%, SU/SCr < 20 (using urea and creatinine mg/dL), and UCr/SCr < 20 indicated renal azotaemia [18,19].

### 2.4. Statistical Analysis

The results were analyzed using the program Statistica 13. The Mann–Whitney *U* test was used to compare the serum activities of AST and ALT, and the de Ritis quotient between groups A and B, and between subgroups A1 and A2. Spearman’s rank correlation coefficient was used to calculate correlations between the de Ritis quotient, serum AST activity, serum ALT activity, and RFI, FE(Na^+^), SU/SCr, and UCr/SCr in azotaemic dogs (all dogs from group A). Spearman’s rank correlation coefficient was also used to calculate correlations between serum AST and ALT activities in all dogs included in this study (groups A and B), in azotaemic dogs (group A), and in non-azotaemic dogs (group B). Correlations between four renal indices used in this study were calculated using Spearman’s rank correlation coefficient (Supplementary Material). A value of *p* < 0.05 was considered significant.

## 3. Results

Azotaemia was identified in 22 infected dogs (group A). In 12 dogs, both serum urea and creatinine concentrations were not elevated above reference intervals (group B). Pre-renal azotaemia was recognized in 13 dogs (sub-group A1), and renal azotaemia was diagnosed in nine infected dogs (subgroup A2). Medians of serum urea and creatinine concentration in groups A and B, and sub-groups A1 and A2 are shown in Table 1.

**Figure 1:**
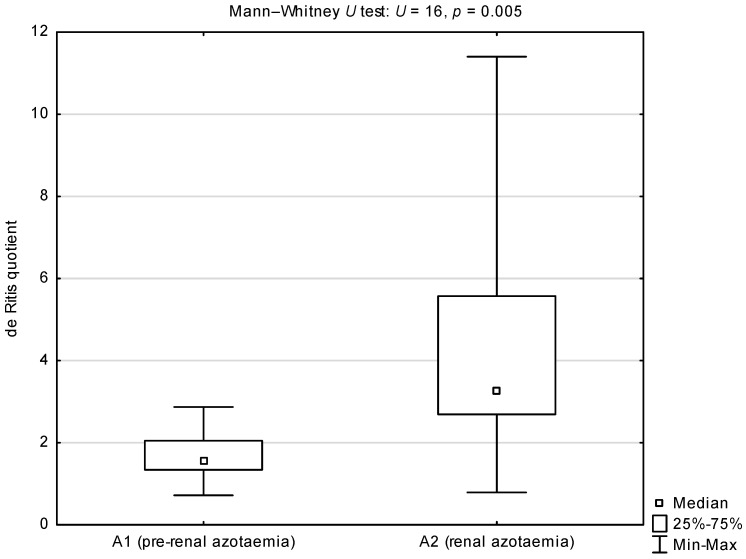
Comparison of the de Ritis quotient (AST/ALT ratio) between azotaemic dogs infected with *B. canis* from sub-group A1 (pre-renal azotaemia) and A2 (renal azotaemia). A1 median and 25–75% interval: 1.54 (1.34–2.05). A1 standard deviation and coefficient of variation: 0.659 and 0.392, respectively. A2 median and 25–75% interval: 3.28 (2.69–5.57). A2 standard deviation and coefficient of variation: 3.089 and 0.691, respectively.

**Figure 2:**
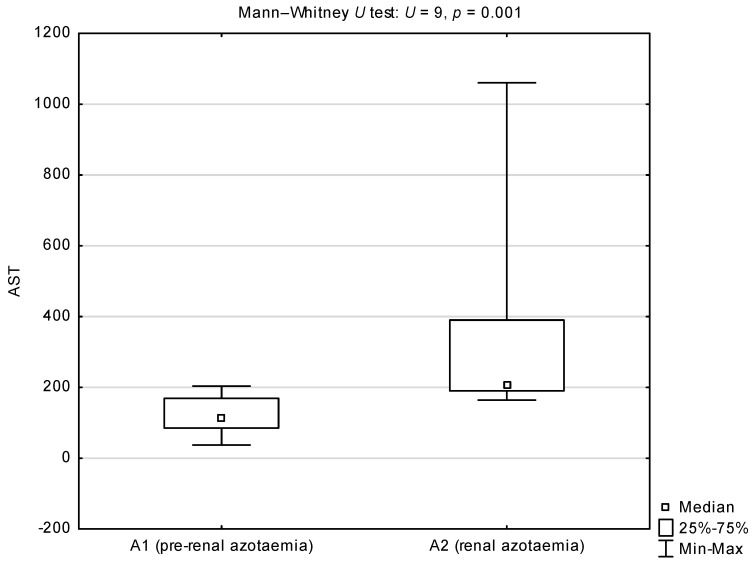
Comparison of serum AST activity (IU/L) between azotaemic dogs infected with *B. canis* from sub-group A1 (pre-renal azotaemia) and A2 (renal azotaemia). A1 median and 25–75% interval: 115 (85–169) IU/L. A1 standard deviation and coefficient of variation: 52.022 and 0.437, respectively. A2 median and 25–75% interval: 206 (190–390) IU/L. A2 standard deviation and coefficient of variation: 285.377 and 0.834, respectively.

**Figure 3:**
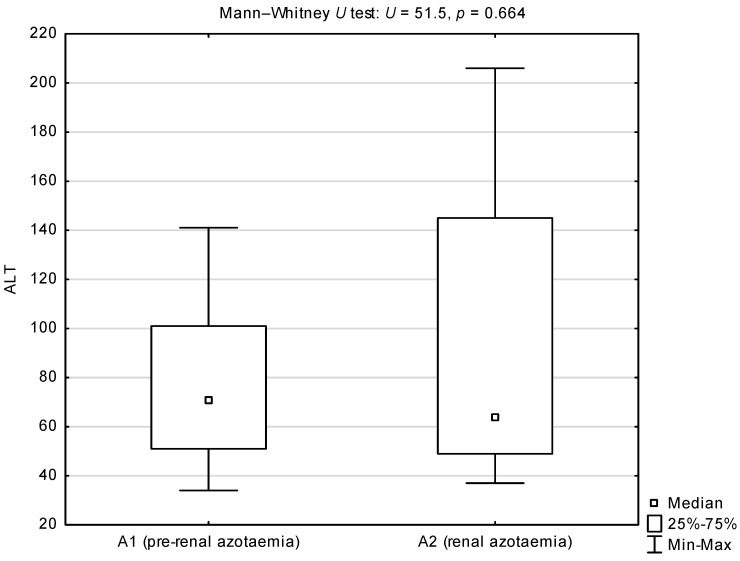
Comparison of serum ALT activity (IU/L) between azotaemic dogs infected with *B. canis* from sub-group A1 (pre-renal azotaemia) and A2 (renal azotaemia). A1 median and 25–75% interval: 71 (51–101) IU/L. A1 standard deviation and coefficient of variation: 37.086 and 0.484, respectively. A2 median and 25%–75% interval: 64 (49–145) IU/L. A2 standard deviation and coefficient of variation: 62.731 and 0.669, respectively.

Comparisons of the de Ritis quotient, and serum activities of AST and ALT between groups A and B showed statistically higher medians for the AST/ALT ratio and AST activity in group A (*p* = 0.002 and *p* = 0.003 respectively; Table 2). There were no differences in serum ALT activity between groups A and B. Comparison of the de Ritis quotient between sub-groups A1 and A2 showed a statistically significant higher median in dogs with renal azotaemia (*p* = 0.005; Figure 1). Comparison of serum AST activity between sub-groups A1 and A2 showed a statistically significant higher median in dogs from sub-group A2 (*p* = 0.001; Figure 2). There were no differences in serum ALT activity between subgroups A1 and A2 (Figure 3).

Statistical analyses also showed significant positive correlations between the de Ritis quotient and renal indices such as RFI and FE(Na^+^) (*p* = 0.013, *p* = 0.012, respectively), and between serum AST activity and the same renal indices (*p* = 0.0002, *p* = 0.00008, respectively) in all azotaemic dogs included in this study (group A). There were also statistically significant negative correlations between the UCr/SCr and the AST/ALT ratio as well as the serum AST activity in these dogs (*p* = 0.032, *p* = 0.0001, respectively). No correlations were observed between SU/SCr and both AST/ALT ratio and serum AST activity. A lack of correlations between serum ALT activity and all renal indices used in this study in dogs from group A was also observed (Table 3).

Serum AST and ALT activities in all 34 dogs from this study (groups A and B combined, *p* = 0.017), as well as in 12 non-azotaemic dogs (group B), were moderately statistically significant and strongly correlated (*p* = 0.009), respectively. However, no correlation between serum AST and ALT activities was observed in the 22 azotaemic dogs from group A (Table 4).

## 4. Discussion

The results of this study are in agreement with the results of previous work examining the association between AST/ALT ratio and azotaemia in canine babesiosis [13]. An increase in serum AST activity and the de Ritis quotient in dogs with renal azotaemia confirms the supposition from previous research that renal damage may cause an elevation in AST activity in canine babesiosis. Moreover, the positive correlations between both AST activity and AST/ALT ratio and renal indices such as RFI and FE(Na^+^) in azotaemic dogs with babesiosis confirms that more severe renal damage leads to greater increases in serum AST activity in infected dogs. Both of these renal indices (RFI and FE(Na^+^)) increase in renal azotaemia; a RFI higher than 2 and FE(Na^+^) higher than 2% indicate renal damage leading to renal azotaemia [19]. Additionally, UCr/SCr ratio below 20 also indicates renal injury [18]. The observation in this study of negative correlations between the UCr/SCr ratio and both the AST/ALT ratio and serum AST activity may also confirm that the kidneys are at least partially a source of increased serum AST activity in canine babesiosis.

The lack of correlations between SU/SCr and both the serum AST activity and AST/ALT ratio may suggest that this renal index is not as useful in canine babesiosis as the other three renal indices used in this study in assessing renal damage in infected dogs. This supposition is supported by the lack of correlations between this parameter and the other three parameters of renal injury (RFI, FE(Na^+^), and UCr/SCr) in all azotaemic dogs from this research, whereas RFI, FE(Na^+^), and UCr/SCr were all statistically significantly correlated with each other (Supplementary Material). It cannot be excluded that the lack of correlation between SU/SCr and AST or the de Ritis quotient may also result from the small number of dogs included in this study. However, a previous study also showed a lack of correlation between SU/SCr and arterial blood pressure in azotaemic dogs with babesiosis, despite hypotension being involved in the development of azotaemia in canine babesiosis [20]. Explanations for this observation can be cardiac injury, hemolysis, or hyperureagenesis caused by ammonia loading as a result of gastrointestinal bleeding, which may lead to increased SU/SCr ratio in canine babesiosis [18,21].

In dogs, hepatocytes are the main source of serum ALT, and serum levels of this enzyme increase during liver injury [22]. This complication of canine babesiosis was observed in previous studies [10,23,24]. Thus, increased serum ALT activity in dogs from this study is not surprising. Skeletal muscles are another potential source of ALT in the blood of dogs, and rhabdomyolysis was observed in dogs infected with *B. rossi* [25,26]. However, this complication of canine babesiosis is rare [27], and to the authors’ best knowledge, has never been described in dogs infected with *B. canis*. Consequently, the lack of difference between the medians of serum ALT activity in azotaemic and non-azotaemic dogs, and in dogs with pre-renal and renal azotaemia, in the context of significant differences in AST activity and AST/ALT ratio between these groups of dogs, also indicates that the kidneys might be a significant source of increased serum AST activity in dogs with renal azotaemia. However, this conclusion needs additional experiments using a histopathological examination of the liver, kidneys, and heart of the infected dogs with determination of the expression of AST and ALT genes in kidney tissue using qPCR.

Moderate correlation between serum AST and ALT activity was observed in all 34 dogs from this study, and strong correlation was observed in the 12 non-azotaemic dogs infected with *B. canis*. These results show that increased serum AST activity is associated with liver injury in canine babesiosis. This observation is obvious, as AST is an enzyme present in the cytosol and mitochondria of hepatocytes [28]. However, the lack of correlation between AST and ALT activities in azotaemic dogs may also indicate that increased serum AST activity did not result solely from liver damage. The authors postulate that significant serum AST activity in infected dogs with kidney injury may be of renal origin. However, it cannot be excluded that increased activity of serum AST may also result from cardiac injury, which is one of the complications in canine babesiosis [10,28,29].

The main limitation of this study is the small number of dogs utilized. However, during clinical observations, the authors have noted decreasing numbers of dogs infected with *B. canis* over recent years. The authors propose that this may stem from the treatment of dogs with the new group of acaricides (isoxazolines), preventing tick infestations over the last few years. These drugs have shown high efficacy at preventing *B. canis* transmission from infected ticks to dogs [30,31]. Moreover, the small number of dogs from this study was limited owing to other diseases or an inability to collect urine sample in one dog. Exclusion of co-infection with *A. phagocytophilum* was deemed to be crucially important in this study owing to the fact that the course of canine granulocytic anaplasmosis may be similar to babesiosis, and increased serum AST activity is the most prevalent biochemical abnormality observed in dogs infected with *A. phagocytophilum* [32].

The AST/ALT ratio may be considered a useful and convenient indicator of renal damage in canine babesiosis. Calculation of this parameter requires only the determination of serum AST and ALT activities, and there is no need to collect urine samples and determine serum and urine parameters, as used in calculations of renal indices such as RFI, FE(Na^+^), and UCr/SCr ratio. However, further study on a larger group of infected dogs is needed. Moreover, determination of other indices of kidney injury, such as kidney injury molecule-1 and neutrophil gelatinase-associated lipocalin, which were detected in canine babesiosis, could allow for the determination of the de Ritis quotient, which indicates renal injury in azotaemic dogs [33,34].

## 5. Conclusions

The results of this study indicate that increased serum AST activity in azotaemic dogs infected with *B. canis* may have a renal origin, and the AST/ALT ratio could be considered as a simple and convenient renal index that is useful in the recognition of renal azotaemia in canine babesiosis.

## Figures and Tables

**Table 1:** Serum urea and creatinine concentrations in dogs infected with *B. canis* from groups A and B, and sub-groups A1 and A2.

Group or Sub-Group	Median (25th–75th Percentile)	
	Urea (mg/dL)	Creatinine (mg/dL)
A (azotaemic)	194.5 (110–311)	3.1 (1.5–4.1)
B (non-azotemic)	41 (36.5–42)	1.25 (1–1.35)
A1 (pre-renal azotaemia)	132 (85–181)	1.8 (1.5–2.9)
A2 (renal azotaemia)	265 (227–341)	5.1 (3.7–6.9)

**Table 2:** Comparisons of the AST/ALT ratio and serum AST and ALT activities between dogs infected with *B. canis* from groups A (azotaemic) and B (non-azotaemic) using Mann–Whitney *U* test.

Parameter	Median (25th%–75th%)		Min–Max		*U*	*p*
	A (Azotaemic)	B (Non-Azotaemic)	A	B		
AST/ALT ratio	2.17 (1.35–3.22)	1.08 (0.77–1.375)	0.72–11.4	0.15–2.54	48	0.002 *
AST (IU/L)	171.5 (95–204)	89.5 (46–118.5)	37–1060	27–159	49.5	0.003 *
ALT (IU/L)	67.5 (49–125)	66.5 (48–91)	34–206	32–1050	125.5	0.828

* statistically significant results. AST: aspartate aminotransferase; ALT: alanine aminotransferase.

**Table 3:** Correlations between renal indices and the de Ritis quotient and serum AST and ALT activities in azotaemic dogs infected with *B. canis*.

Correlations		*R*	*p*
De Ritis quotient	RFI	0.520	0.013 *
	FE(Na^+^)	0.522	0.012 *
	SU/SCr	0.074	0.74
	UCr/SCr	−0.460	0.032 *
AST	RFI	0.715	0.0002 *
	FE(Na^+^)	0.739	0.00008 *
	SU/SCr	−0.081	0.721
	UCr/SCr	−0.732	0.0001 *
ALT	RFI	0.143	0.526
	FE(Na^+^)	0.186	0.407
	SU/SCr	−0.031	0.891
	UCr/SCr	−0.282	0.203

* statistically significant results. RFI: renal failure index; FE(Na^+^): sodium fractional excretion; SU/SCr: serum urea to creatinine ratio; UCr/SCr: urinary creatinine to serum creatinine ratio.

**Table 4:** Correlations between serum AST and ALT activities in 34 dogs infected with *B. canis* (groups A and B combined), 22 azotaemic dogs (group A), and 12 non-azotaemic dogs (group B).

Correlation	*R*	*p*
Groups A and B combined	0.407	0.017 *
Group A (azotaemic)	0.296	0.181
Group B (non-azotaemic)	0.714	0.009 *

* statistically significant results.

## Data Availability

The data presented in this study are available in this paper.

## Supplementary Materials

The following are available online at https://www.mdpi.com/article/10.3390/ani12050626/s1: Table S1: Correlations between renal indices in 22 azotaemic dogs infected with *B. canis* (group A).
