# Supplementary material for: Association between Increased the De Ritis Quotient and Renal Azotaemia in Canine Babesiosis
Source: Animals (Basel). 2022 Mar 2;12(5):626. doi: 10.3390/ani12050626 (PMC8909065; doi:10.3390/ani12050626)
Supplement: Supplementary file 1 [file animals-12-00626-s001.zip › animals-1571395-supplementary.pdf]

Supplementary material

PCR details:
Babesia canis (used to amplify the 18S ribosomal DNA gene fragment of B. canis):
Primers:
Forward primer (BcW-A): 5'-CAT CTA AGG AAG GCA GCA GG-3'
Reverse primer (BcW-B): 5'-TTA ATG GAA ACG TCC TTG GC-3'
PCR mixture (50 µL):
10 µM both primers (1 µL of each primer),
10 mM dNTP Mix (2 µL),
10× Taq Buffer with KCl (5 µL),
2 IU of Taq DNA Polymerase (1 UI/1µL),
25 mM $MgCl_2$ (4 µL),
extracted template DNA (10 µL),
injection water (25 µL)
PCR:
Preamplification denaturation 94°C (2 min)
36 cycles:
Denaturation 94°C (30 s)
Annealing 53 °C (30 s)
Elongation 72 °C (30 s)
Final elongation 72 °C (5 min)
The size of the PCR products about 500 bp (electrophoresis in a 1.5% agarose gel)

Anaplasma phagocytophilum (used to amplify *epank1* gene fragment of A. phagocytophilum)
Primers:
Forward primer (LA6): 5′-GAG AGA TGC TTA TGG TAA GAC-3′
Reverse primer (LA1): 5′-CGT TCA GCC ATC ATT GTG AC-3′
PCR mixture (50 µL):
10 µM both primers (1 µL of each primer)
10 mM dNTP Mix (2 µL),
10× Taq Buffer with KCl (5 µL),
2 IU of Taq DNA Polymerase (1 UI/1µL),
25 mM $MgCl_2$ (4 µL),
extracted template DNA (10 µL),
injection water (25 µL)
PCR:
Preamplification denaturation 94°C (2 min)
2 cycles:
Denaturation 94°C (30 s)
Annealing 62 °C (30 s)
Elongation 72 °C (30 s)
2 cycles:
Denaturation 94°C (30 s)
Annealing 60 °C (30 s)
Elongation 72 °C (30 s)
2 cycles:
Denaturation 94°C (30 s)
Annealing 58 °C (30 s)
Elongation 72 °C (30 s)
2 cycles:
Denaturation 94°C (30 s)

Annealing 56 °C (30 s)
Elongation 72 °C (30 s)

28 cycles:

Denaturation 94°C (30 s)
Annealing 54 °C (30 s)
Elongation 72 °C (30 s)

Final elongation 72 °C (5 min)

The size of the PCR products about 440 bp (electrophoresis in a 1.5% agarose gel)

Figure S1. Babesia canis merozoites detected in microscopic blood smear examination

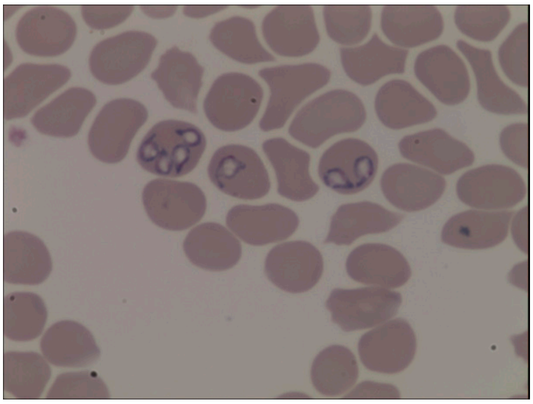

Figure S2. Results of B. canis PCR (Eurx Perfect™ 100-1000 bp DNA laddder), PCR product 500 bp.

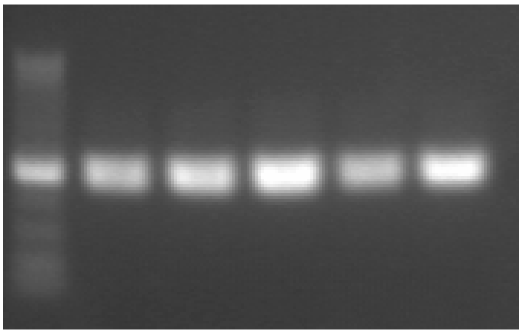

Azotaemia (group A) in 22 out of 34 dogs (64.71%; Confidence interval: 46.49% - 80.25%)

Lack of azotaemia (group B) in 12 out of 34 dogs (35.29%; Confidence interval: 19.75% - 53.51%)

Pre-renal azotaemia (subgroup A1) in 13 out of 22 azotaemic dogs (59.09%; Confidence interval: 36.35% - 79.29%)

Renal azotaemia (subgroup A2) in 9 out of 22 azotaemic dogs (40.91%; Confidence interval: 20.71% - 63.64%).

Table S1. Correlations between renal indices in 22 azotaemic dogs infected with *B. canis* (group A)

	RFI	FE(Na^+)	UCr/SCr	SU/SCr
RFI		R = 0.991, p = 0.000*	R = -0.919, p = 0.000*	R = -0.318, p = 0.149
FE(Na^+)	R = 0.991, p = 0.000*		R = -0.924, p = 0.000*	R = -0.308, p = 0.163
UCr/SCr	R = -0.919, p = 0.000*	R = -0.924, p = 0.000*		R = 0.361, p = 0.098
SU/SCr	R = -0.318, p = 0.149	R = -0.308, p = 0.163	R = 0.361, p = 0.098	

*statistically significant result
